# Extracorporeal Membrane Oxygenation as Treatment of Severe COVID-19 Infection: A Case Report

**DOI:** 10.7759/cureus.7714

**Published:** 2020-04-17

**Authors:** Steven Douedi, Abbas Alshami, Eric Costanzo

**Affiliations:** 1 Internal Medicine, Hackensack Meridian Health, Jersey Shore University Medical Center, Neptune, USA; 2 Internal Medicine, Dorrington Medical Associates, Houston, USA; 3 Pulmonology and Critical Care, Hackensack Meridian Health, Jersey Shore University Medical Center, Neptune, USA

**Keywords:** covid-19, coronavirus, ecmo, ards, respiratory, infection

## Abstract

Novel coronavirus 2019 (COVID-19) is a severe respiratory infection leading to acute respiratory distress syndrome (ARDS) accounting for thousands of cases and deaths across the world. Several alternatives in treatment options have been assessed and used in this patient population. However, when mechanical ventilation and prone positioning are unsuccessful, venovenous extracorporeal membrane oxygenation (VV-ECMO) may be used.

We present a case of a 41-year-old female, with no significant medical history and no recent history of exposure to sick contacts, presented to the emergency department (ED) with fever, severe shortness of breath, and flu-like symptoms with a positive COVID-19 test. Ultimately, she worsened on mechanical ventilation and prone positioning and required VV-ECMO.

The use of VV-ECMO in COVID-19 infected patients is still controversial. While some studies have shown a high mortality rate despite aggressive treatment, such as in our case, the lack of large sample size studies and treatment alternatives places healthcare providers against a wall without options in patients with severe refractory ARDS due to COVID-19.

## Introduction

The novel coronavirus 2019 (COVID-19) is a respiratory tract infection that has resulted in a pandemic, infecting more than 1,250,000 humans and claiming the lives of over 75,000 in less than six months [1]. The disease classically results in hypoxemic respiratory failure requiring oxygen supplementation using low and high delivery systems, as well as mechanical ventilation. However, when all these measures fail, options become very limited. One of these potential alternatives is the extracorporeal membrane oxygenation (ECMO). Evidence on ECMO in COVID-19 patients remains controversial, as the immunological side effects of ECMO can further compromise the already debilitated immune system fighting COVID-19 [2]. We report a case of a COVID-19-positive patient who was managed with ECMO after no response to mechanical ventilation and prone positioning.

## Case presentation

**Figure 1:**
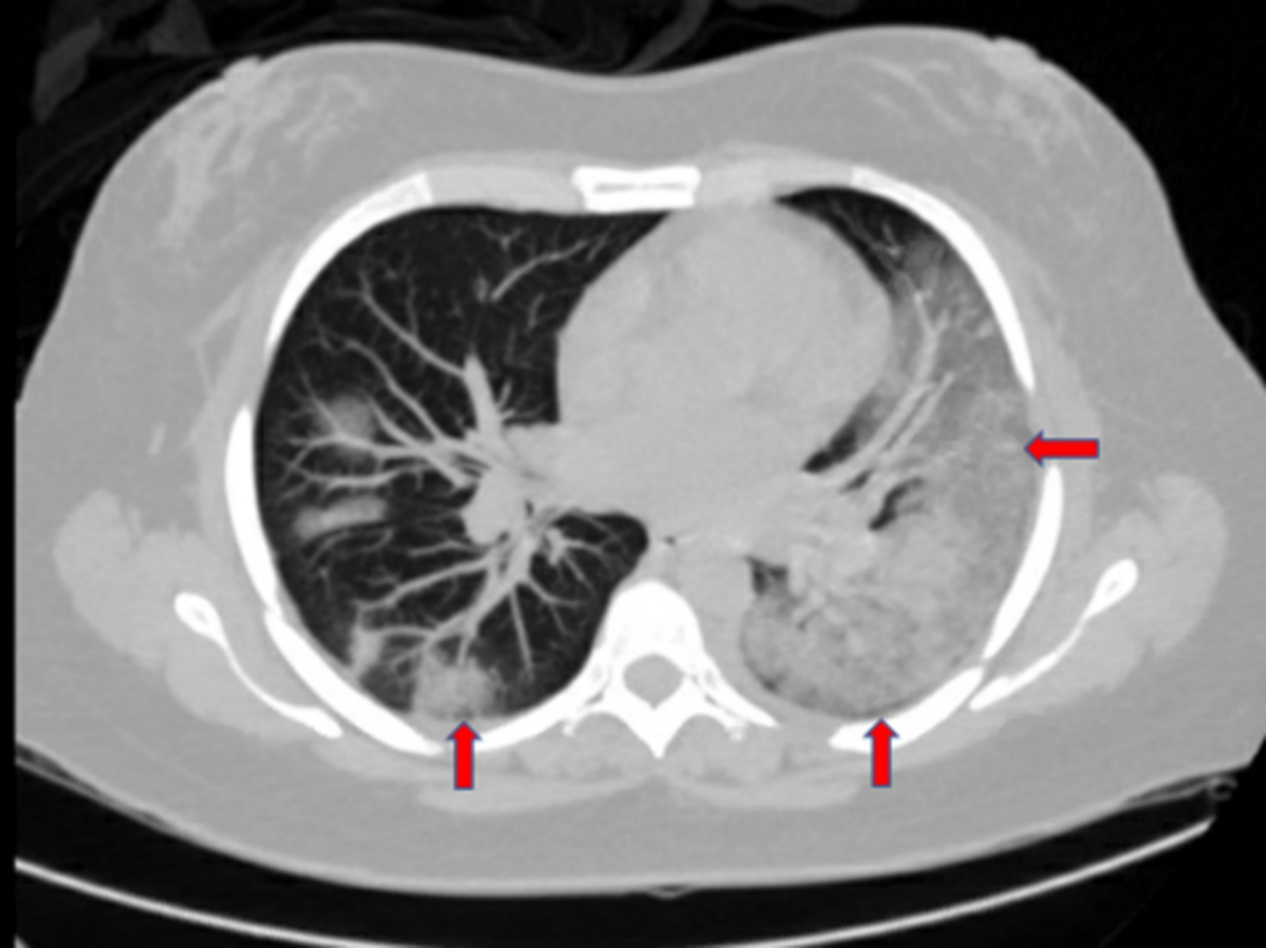
CT scan of the chest without contrast Bilateral diffuse scattered patchy ground-glass opacities throughout the lungs with more geographic mixed ground-glass and consolidative opacities in the lingular and superior segment of the left lower lobe extending to the posterior left lower lobe. Mild to moderate patchy scattered ground-glass opacities were seen in the right lower lobe, as well as a perihilar right upper lobe with areas of peripheral ground-glass opacities

A 41-year-old female with no significant medical history and no recent history of travel or exposure to sick contacts presented to the emergency department (ED) with a worsening dry cough, shortness of breath, and chest tightness, followed by fever, chills, and myalgias for four days duration. Other reported symptoms included a mild sore throat and watery diarrhea. Vital signs on admission were a temperature of 104.2°Fahrenheit (measured orally), a heart rate of 120 beats per minute, a blood pressure of 130/62 mm Hg, respiratory rate of 32 breaths per minute, and oxygen saturation of 89% on room air (94% on 2 liters nasal cannula). Physical examination was pertinent for ill appearance and rhonchi over the left lung base. Blood tests showed a white blood cell count of 11.7 cells/mm3, lymphopenia of 700 cells/mm^3^, hemoglobin of 11.6 g/dL, potassium of 2.8 mEq/L, aspartate aminotransferase (AST) of 85 IU/L, alanine aminotransferase (ALT) of 66 IU/L, lactate of 1.4 mmol/L, and procalcitonin 1.57 ng/mL. Polymerase chain reaction (PCR) tests for influenza A and B, metapneumovirus, adenovirus, parainfluenza, respiratory syncytial virus, and coronaviruses HKU1, NL63, 229E, and OC43 were all negative. Given the current pandemic, COVID-19 was suspected, and a nasal swab was sent to be tested. A computed tomography scan of the chest was obtained and showed bilateral infiltrates (Figure 1).

The patient was started empirically on intravenous (IV) vancomycin, piperacillin-tazobactam, azithromycin, and hydroxychloroquine. Over a one-day period, the patient’s respiratory status progressively deteriorated, and she was subsequently intubated. On the following day, the COVID-19 test came back positive, and the patient was continued on 400 mg daily of hydroxychloroquine and 500 mg twice daily of azithromycin. She was also started on high-dose vitamin C at a rate of 6 grams IV twice daily, and 220 mg of zinc sulfate via orogastric tube once daily. Despite aggressive management, she developed severe acute respiratory distress syndrome (ARDS) and was requiring higher mechanical ventilation settings (100% fraction of inspired oxygen and 16 of positive end-expiratory pressure). The decision was also made to begin prone positioning of the patient for 18 hours a day for a ratio of arterial oxygen partial pressure to fractional inspired oxygen (P/F ratio) of < 100. Liver enzymes continued to trend up (AST 274 and ALT 300), and the patient developed acute kidney injury due to decreased organ perfusion. She was started on Levophed for hemodynamic stability and to maintain a mean arterial pressure > 65. She was also given one dose (8 mg/kilogram body weight) of tocilizumab, an anti-interleukin-6 receptor monoclonal antibody, in order to help control her cytokine storm. Despite this, she continued to decompensate, and the patient was started on continuous venovenous hemodialysis (CVVHD) for renal failure and on venovenous extracorporeal membrane oxygenation (VV-ECMO). Prior to VV-ECMO, an echocardiogram was performed which showed an ejection fraction of 60% - 65%, moderate pulmonary hypertension, and grade 1 (mild) diastolic dysfunction. Two days after starting VV-ECMO, the patient lymphocyte count was 0 cells/mm^3^, white blood cell count was 26.1 cells/mm^3^, fibrinogen level < 35, and D-dimer 116,193. She was started on Lovenox, 1 mg/kg, due to a severely elevated D-dimer; however, her platelet count decreased by greater than 50%, and she was switched to Argatroban. Heparin-induced thrombocytopenia (HIT) panel was sent and returned negative, but she remained on Argatroban for anticoagulation due to the significant drop in her platelet count on heparin products. She began to develop ischemia in her fingers and toes bilaterally but was continued on Levophed for hemodynamic stability and VV-ECMO. Four days after the initiation of VV-ECMO, the patient developed an asystole rhythm and ultimately passed away.

## Discussion

Extracorporeal membrane oxygenation (ECMO) has remarkably progressed over recent years and became a reliable tool in severe cardiac and pulmonary dysfunction [3-4]. Venovenous ECMO (VV-ECMO) can be considered in patients with a PF ratio of 70 - 80 mm Hg, Murray score > 3, and a pH of < 7.2 on arterial blood glass [3]. VV-ECMO allows deoxygenated blood to be pulled from the right atrium through a cannula allowing it to pass through an oxygenator and heat exchanger before being pumped back into the right atrium through another cannula [3, 5]. There are no relative contraindications to VV-ECMO as the decision is made on a case-by-case basis; however, the patient’s age and comorbidities must be taken into consideration and an echocardiogram should be performed prior to initiation to evaluate for right or left ventricular failure to confirm the nature of pulmonary failure [3, 5]. Complications of VV-ECMO include bleeding, infection, air embolism, heparin-induced thrombocytopenia (HIT), and catheter/machine-associated dysfunction [5]. Despite these complications, some studies have shown that VV-ECMO significantly improves survival in severe acute respiratory failure, including patients with influenza A (H1N1)-related acute respiratory distress disease [4-6]. Vaquer et al. reported that 60% of patients who received VV-ECMO were successfully discharged from the hospital despite severe refractory ARDS [7]. 

The use of ECMO in COVID-19 patients is still controversial and has mixed results. Li et al. reported seven COVID-19 infected patients with P/F ratios < 100 on VV-ECMO and was able to successfully wean three patients thus far; however, they had a mortality rate of 50% [8]. Yang et al. had similar results where five of six patients receiving ECMO for COVID-19 infection died [9]. It was found that a decreased lymphocyte count was associated with poor outcomes and death from COVID-19 infections [2, 9]. In our case presented, our patient did not respond to mechanical ventilation. Due to a lack of alternatives, her young age, and no comorbidities, VV-ECMO was considered in our patient with severe ARDS (P/F ratio < 100) due to the COVID-19 infection. Ultimately, her lymphocyte count was 0 cells/mm^3^ and she did not respond to VV-ECMO and passed away. While most studies lack a significant sample size, this case adds to the concern on the use of ECMO in COVID-19 patients. In patients with severe ARDS unresponsive to mechanical ventilation, prone positioning, and other alternatives, the need for further studies and understanding the role of ECMO in respiratory failure need to be assessed. 

## Conclusions

VV-ECMO use in patients with severe refractory ARDS due to COVID-19 infections is still controversial. While some studies have shown a high mortality rate despite aggressive treatment, such as in our case, they lack sufficient sample sizes. Due to limited alternatives and treatment options for patients with severe refractory ARDS, studies evaluating the use of ECMO in COVID-19 are desperately needed.
